# Knowledge, attitudes, and behaviors regarding HIV among first time attenders of voluntary counseling and testing services in Italy

**DOI:** 10.1186/1471-2334-13-277

**Published:** 2013-06-19

**Authors:** Gabriella Di Giuseppe, Alessandra Sessa, Silvana Mollo, Natascia Corbisiero, Italo F Angelillo

**Affiliations:** 1 Department of Experimental Medicine, Second University of Naples, Via Luciano Armanni, 5, 80138, Naples, Italy

**Keywords:** Attitudes, Behavior, Human Immunodeficiency Virus, Knowledge, Voluntary counseling and testing

## Abstract

**Background:**

This study assess knowledge, attitudes, and practices regarding Human Immunodeficiency Virus (HIV) testing and counseling services and the predictor characteristics of these outcomes among individuals who presented for the first time to Voluntary Counseling and Testing (VCT) public services.

**Methods:**

A sample of 244 subjects in the geographic area of Naples (Italy) received a self-administered anonymous questionnaire about socio-demographic characteristics, knowledge, attitudes relating to HIV infection, and practices relating to access to VCT service.

**Results:**

Only 25% correctly identified the main modes of transmission and the main preventative measures of HIV and this knowledge was significantly higher in who had had more than one sexual partner and have not always used a condom during the intercourse in the last year, in those who have received information about HIV/AIDS through physician, and in those who have received middle school or lower educational level. The perceived risk of contracting HIV/AIDS was significantly higher in respondents of lower age, in those who perceived a better personal health status, and in those unmarried. Only 20.9% reported that they had received the HIV test and males and those who visited a physician or participated in preventive activities about HIV/AIDS were significantly more likely to have had an HIV test.

**Conclusions:**

This study supports the need to disseminate information and interventions to this population.

## Background

Human Immunodeficiency Virus (HIV) voluntary counseling and testing (VCT) services is internationally well recognized to be one of the most effective public health interventions for both treatment and primary and secondary prevention of this infection. Indeed, VCT relies mostly on individuals presenting themselves for HIV testing, giving voluntary informed consent, and provide an opportunity for individuals to understand their personal risk for HIV infection, make informed choices based on knowledge of their serostatus, encourage change/elimination unhealthy behaviors in order to reduce the risk of becoming infected, assist HIV positive individuals in preventing the transmission of the infection, and obtain medical care if they are HIV-positive. Moreover, HIV testing has been considered a cost-effective procedure in different countries area’s [1-4].

In Italy, 5,8 new HIV infections per 100,000 people were diagnosed in 2011 with an higher prevalence among male and mainly attributable to unsafe sex [5] and, as in many other industrialized and developing countries worldwide, VCT is an important component of the national HIV/AIDS preventive strategies for uninfected people, and for treatment, care efforts, and support for HIV-infected persons [6]. In order to achieve these goals, it is important that participants should be adequately educated on the benefits of the VCT, both in preventing the spread of the HIV infection and meeting the need for care and support in communities.

Very few surveys have evaluated the level of knowledge, the attitudes, and the practices towards VCT among individuals who accessed these services [7-10], seemingly studies have not been done to explore similar questions in Italy. Thus, there is a need for additional studies because an understanding of these topics is crucial for the development of interventions for the disease control, since misconceptions may contribute to prevent individuals from making right choices, decisions, and taking appropriate prevention strategies. The purposes of the current epidemiological survey among individuals who presented for the first time to the VCT public services in Italy were to: (1) conduct a detailed exploration regarding the knowledge, the attitudes, and the practices regarding HIV testing and counseling services; and (2) identify the predictor characteristics of self-reported knowledge about HIV infection, fear of contracting HIV/AIDS, and having received the HIV test.

## Methods

A cross-sectional survey design was used in this study. The eligible study population included all subjects age 18 and older who presented for the first time to seven randomly selected VCT public services in the geographic area of Naples, in the South of Italy, during the period comprised from June 2007 through October 2010. In Italy, the VCT services offers free HIV testing and the service is provided by staff members generally composed of physicians, psychologists, and nurses trained and experienced [11].

All subjects who accessed for the first time to the VCT were invited to participate. They received an information sheet that explained the survey aims and procedures that the participation was completely voluntary and anonymous, that privacy and confidentiality would be strictly protected as no personal identifiers were included in the questionnaire. Individual written informed consent was obtained from all study participants prior to receive the questionnaire. Those who agreed to participate were given the questionnaire to complete and deposit in a sealed box that was designated for that purpose.

All survey questions in the questionnaire were self-reported and included information regarding the following items: (i) the first part contained questions regarding socio-demographic characteristics, and about health status; (ii) in the second part, participants were asked a series of questions measuring their knowledge about HIV infection (modes of transmission and preventive measures); (iii) the third section assessed participants’ attitudes towards the fear of acquiring or transmitting HIV/AIDS, and beliefs if HIV/AIDS was preventable; (iv) in the fourth part, individuals were asked about their health-promoting behavior with questions about HIV sexual risk behaviors (number of partners and number of sexual intercourses and how frequently they were protected by condoms during acts of intercourse with the partner(s) in the last year), injecting drugs use in the last year, frequency of sharing needles in the last year, frequency of a health checkup in the last year, reasons for access to VCT service, whether respondents’ health care provider informed about VCT service, and whether they have received the HIV test; and (v) in the final section, participants were asked if they have family’s/friend’s history of HIV/AIDS and if they have accessed to the VCT, their main sources of and needs of information regarding HIV/AIDS.

Self-rated health status was assessed on a ten-point Likert-type scale, with responses ranging from 1 (poor) to 10 (excellent). For items regarding the knowledge, participants were asked to provide answers with options for “no”, “do not know”, and “yes” and to indicate using a 3-point Likert-type scale whether or not they agreed or disagreed with statements. The responses of the question measuring the attitudes were on 3-point Likert-type scale in the format “agree”, “uncertain”, and “disagree” and on a Likert scale with a score ranging from 1 to 10 with higher scores indicated higher perceptions about their risk of transmitting and contracting HIV/AIDS. The respondents were also asked to report directly reason(s) for have or have not received HIV test. Questions pertaining to behaviors were close ended with nominal or categorical (yes or no) responses; the frequency of use of condoms in the last year was measured on a five-point Likert scale ranging from “never” to “every time”.

The questionnaire was tested in a pilot study on a convenience sample of 20 adults, the test respondents commented on some questions, and subsequently the wording and order of some questions were made before the final version was completed.

The study protocol and survey instrument were approved by the Ethic committee of the Second University of Naples.

### Statistical analysis

The statistical analyses were executed in two steps. First, the effect of the several characteristics and the outcomes of interest were observed using appropriate bivariate analysis such as chi-square test for categorical variables and t-test for continuous variables. Then, multivariate linear and logistic regression were performed, entering variables when the *p*-value of the bivariate association was ≤ 0.25, for identifying the factors that were independently associated with the different outcomes of interest and variables were selected for multivariate models with *p*-value < 0.2 for entry and *p*-value < 0.4 for exclusion. The main outcomes of interest were defined as (i) knowledge about the modes of transmission and the main preventative measures of HIV (no = 0, yes = 1); (ii) fear of contracting HIV/AIDS (continuous); and (iii) having received the HIV test (no = 0, yes = 1). All models included the following variables: gender (male=0, female = 1), age (continuous, in years), marital status (married = 1, single/separated/divorced/widowed=0), having a child (no = 0, yes = 1), educational level (three categories: middle school or lower = 1, high school = 2, baccalaureate degree/graduate degree = 3), employment status (unemployed = 0, employed = 1), number of other persons in the household (0 = 0, ≥1 = 1), perception of personal health status (continuous), having had more than one sexual partner and no consistent condom use in the last year (no = 0, yes = 1), injecting drugs use in the last year (no=0, yes=1), at least one member in the household/close friends having history of HIV/AIDS (no = 0, yes = 1), physician as source of information about HIV/AIDS (no = 0, yes = 1), and need of additional information about HIV/AIDS (no = 0, yes = 1). The following variables were also included: having received the HIV test (no = 0, yes = 1) in Model 2; at least one familiar/friend have accessed to VCT (no = 0, yes =1), and knowledge about the modes of transmission and the main preventative measures of HIV (no = 0, yes = 1) in Models 2 and 3; fear of contracting HIV/AIDS (continuous), fear of transmitting HIV/AIDS (continuous), HIV may be prevented (no = 0, yes = 1), and having visited a physician or having participated in preventive activities about HIV/AIDS (no = 0, yes = 1) in Model 3. The strength of association between the dependent variables with the outcome of interest is presented using odds ratios (ORs), while controlling for the other variables in the multivariate logistic regression models, with their 95% confidence intervals (CIs). Standardized regression coefficients (β) were presented in the multivariate linear regression model. Determinants were reported to be significantly associated when they had a *p*-value of 0.05 or lower. All *p*-values were two-tailed. Analysis of data was performed using the computer software Stata, 2009 [12].

## Results

A total of 301 individuals were recruited and 244 agreed to participate in the study for a response rate of 81.1%. Table 1 provides an overview of the respondents socio-demographic and general characteristics. The average age was 33.6 years, one-third was female, the majority had completed a high school education, more than one-third had at least one member in the household/close friends having history of HIV/AIDS, 25% had a familiar/friend who have accessed to VCT, and the overall mean score for perceived personal health status was 7.2. In the previous year, 14.3% reported injecting drug use and less than half have shared needles. Regarding the sexual behavior pertinent to the transmission of HIV, 87.3% had sexual intercourse in the last year, and among the subsample of sexually active participants in the last year only 12.3% reported that they had always used the condom during the intercourse with casual or regular partner.

**Table 1:** Principal characteristics and HIV risk behavior among participants

	***N***	***%***
*Gender*		
Male	165	67.6
Female	79	32.4
*Age group (years)*	33.6 ± 11.4 (17–69)*	
<25	64	26.2
25-29	51	20.9
30-34	29	11.9
35-39	30	12.3
40-44	22	9
≥45	48	19.7
*Marital status*		
Married	169	69.3
Single/separated/divorced/widowed	75	30.7
*Number of other persons in the household*		
0	51	20.9
1	18	7.4
2	53	21.7
3	55	22.5
>3	67	27.5
*Highest educational level*		
No formal education/Elementary school	13	5.3
Middle school	49	20.1
High school	128	52.5
Baccalaureate degree/Graduate degree	54	22.1
*Employment status*		
Employed	139	57
Unemployed	105	43
*Number of child*		
0	161	66
1	31	12.7
2	35	14.3
≥3	17	7
*Perception of personal health status*	7.2 ± 1.9 (1–10)*	
*At least one member in the household/close friends having history of HIV/AIDS*		
No	148	60.7
Yes	96	39.3
*At least one familiar/friend who have accessed to VCT*		
No	183	75
Yes	61	25
*Number of sexual partners in the last year*		
0	31	12.7
1	71	29.1
2	42	17.2
≥3	100	41
*Condom use in the last year*^*+ a*^		
Never	77	36.5
Rarely	33	15.6
Sometime	36	17.1
Often	39	18.5
Always	26	12.3
*Injecting drug use in the last year*		
No	209	85.7
Yes	35	14.3
*Shared needles*^*++*^		
No	18	51.4
Yes	17	48.6

^*^Mean±Standard deviation (Range).

^+^Only for those who had sexual intercourse in the last year (213).

^++^Only for those who had inject drug in the last year (35).

^a^The number of participants responding to this question is 211.

The respondents’ knowledge about HIV infection is reported in Table 2. The analysis of the different questions showed that the frequencies of the correct answers ranged from 21.3% to 92.2% regarding the modes of HIV transmission and from 64.7% to 95.9% of the specific means of preventive measures. Overall, only 25% correctly identified the main modes of transmission and the main preventative measures of HIV. The results of the multivariate regression analysis are shown in Table 3, which illustrates factors associated with the different outcomes of interest. Multiple logistic regression indicated that three characteristics significantly influenced the level of knowledge. Indeed, the respondents who had more than one sexual partner and have not always used a condom during the intercourse in the last year (OR=0.33; 95% CI 0.16-0.7), those who have received information about HIV/AIDS through physician (OR = 4.34; 95% CI 2.01-9.42) were more likely to correctly answer the questions about the modes of transmission and the main preventative measures of HIV. Moreover, when the baccalaureate degree/graduate degree was chosen as reference category, a value significantly lower than 66% was observed for those who have received middle school or lower educational level (95% CI 0.14-0.84) (Model 1 in Table 3).

**Table 2:** Knowledge of the study population toward HIV

	***Correct response***	
	***N***	***%***
*Modes of transmission*		
Heterosexual intercourse without a condom (*True*)	225	92.2
Needle sharing (*True*)	220	90.2
Homosexual intercourse without a condom (*True*)	198	81.1
Tattoos and piercing with contaminated needles (*True*)	161	66
Vaginal delivery (*True*)	89	36.5
Breastfeeding (*True*)	52	21.3
*Preventive measures*		
Use of condom during acts of intercourse (*True*)	234	95.9
Avoiding sharing needle (*True*)	226	92.6
Avoiding sharing fork or glasses (*False*)	192	78.7
Bottle feeding (*False*)	173	70.6
Avoiding sharing sharp personal objects (*True*)	158	64.7

**Table 3:** Multivariate logistic (1 and 3) and linear (2) regression models results

**Variable**	**OR**	**SE**	**95% CI**	***p *value**
**Model 1.** Knowledge about the modes of transmission and the main preventative measures of HIV (Sample size=244)				
Log likelihood=−118.4, χ^2^=37.67 (6 df), *p*<0.0001				
Physician as source of information about HIV/AIDS	4.34	1.71	2.01-9.42	<0.001
Having had more than one sexual partner and no consistent condom use in the last year	0.33	0.13	0.16-0.7	0.004
Educational level				
Middle school or lower	0.34	0.16	0.14-0.84	0.019
Baccalaureate degree/graduate degree*	1.0			
Employment status	0.63	0.2	0.33-1.19	0.15
Need of additional information about HIV/AIDS	0.63	0.21	0.33-1.21	0.17
Number of other persons in the household	0.69	0.27	0.32-1.47	0.34
**Model 3.** Having received the HIV test (Sample size=244)				
Log likelihood=−114.71, χ^2^=20.76 (5 df), *p*<0.0001				
Having visited a physician or having participated in preventive activities about HIV/AIDS	2.67	0.93	1.35-5.29	0.005
Gender	0.42	1.66	0.19-0.91	0.028
Knowledge about the modes of transmission and the main preventative measures of HIV	1.73	0.62	0.85-3.52	0.13
Educational level				
Middle school or lower	0.54	0.23	0.23-1.26	0.15
Baccalaureate degree/graduate degree*	1.0			
Injecting drugs use in the last year	1.85	0.8	0.79-4.33	0.16
**Variable**	**Coeff**	**SE**	***t***	***p *value**
**Model 2.** Fear of contracting HIV/AIDS (Sample size=244)				
F(6,237)=6.96, *p*<0.0001, R^2^=15%, adjusted R^2^=12.8%				
Perception of personal health status	0.25	0.08	2.94	0.004
Age	−0.04	0.02	−2.48	0.014
Marital status	−0.84	0.41	−2.07	0.04
Need of additional information about HIV/AIDS	0.5	0.33	1.5	0.13
Gender	0.51	0.34	1.5	0.13
Having received the HIV test	0.39	0.39	1.01	0.31
Constant	5.87			

*Reference category.

The answers regarding the attitudes towards HIV/AIDS indicated that approximately three-quarters (71.7%) of the respondents thought that was possible to prevent HIV/AIDS, that the level of perceived risk of transmitting and contracting HIV/AIDS resulted in a mean total score respectively of 6±3 and 6.6±2.6, and a high perceived risk was indicated by 17.6% and 18.8% who responded 10 to the questions. A stepwise multiple linear regression was conducted to assess which variables predicted the perception of risk of contraction HIV/AIDS. The perceived risk was significantly higher in respondents of lower age, in those who perceived a better personal health status, and in those unmarried (Model 3 in Table 3).

The analysis of the results showed that the most commonly cited reasons for seeking VCT were for learning about their HIV sero-status (49.5%), own or partners’ high risk sexual behaviors (39.1%), and specific symptoms of HIV/AIDS (6.6%). A total of 51 subjects (20.9%) reported that they had received the HIV test and almost three-quarters (74.4%) reported voluntary HIV test. In the final logistic regression model two variables were significantly associated with having tested for HIV. Males (OR=0.42; 95% CI 0.19-0.91) and those respondents who visited a physician or participated in preventive activities about HIV/AIDS (OR=2.67; 95% CI 1.35-5.29) were significantly more likely to have had an HIV test (Model 2 in Table 3). Among those who had been tested, the main reasons were blood donation (20.9%), engaged in high risk sexual behaviors (16.3%), and wanting reassurance of being uninfected (7%); whereas, the most common deterrent from going for an HIV test were fear (48.3%), and that they feel to be not at risk (44.8%).

When asked about their main source of information about HIV/AIDS, a substantial proportion of the participants indicated that they have received information (92.2%). Most respondents reported that mass media were the major sources of their information (73.8%), followed by scientific journals (24%), and only 18.2% claimed physicians. The vast majority (64.3%) expressed an interest in receiving additional information.

## Discussion

To our knowledge, this study is one of the limited number of published investigations that has rigorously addressed individuals’ knowledge, attitudes, and practices towards VCT among those who accessed for the first time to the VCT.

One of the main finding of this study was that only 25% of current participants were able to correctly identify the HIV main modes of transmission and the main preventative measures. Many more participants were able to respond to some questions; namely, more than 90% of participants identified as modes of transmission the heterosexual intercourse without a condom and the needle sharing and identified the correct preventive measures the use of condom during acts of intercourse and avoiding sharing needle.

The findings from the present study suggest that there were several socio-demographic characteristics of the study population that were associated with the different outcomes of interest. Indeed, there were significant differences between individuals with different levels of education and knowledge about AIDS. In the logistic regression model regarding the level of knowledge, as expected, those with higher educational levels, especially those with a university or college education, were more likely to correctly answer the questions about the modes of transmission and the main preventative measures of HIV. A risky sexual behaviors were independently associated with the level of knowledge, since those who had sex with more than one sexual partners and have not always used a condom during the intercourse in the last year were more knowledgeable. These findings not only highlight the high level of risk behaviors, but also indicate that prevention researchers may be better equipped to design interventions to minimize and possibly change high risk behaviors. Another interesting finding from this survey was the fact that younger and unmarried respondents perceived to be at higher risk of contracting HIV/AIDS. One of the most intriguing result from this study was that respondents who reported a better perceived personal health status were more likely to perceive a higher risk of contracting HIV/AIDS. A reason is offered as possible explanation for this finding that healthy subjects may place more importance on maintaining their health. Furthermore, males were also found to be more likely to perform HIV test than females.

In this sample, the majority of the respondents acquired their knowledge about HIV/AIDS from mass media. This result is in accordance to another similar published study [9]. Despite television was the most frequently reported source, they appear to be not effective for retaining knowledge, whereas very little information was obtained from health care providers where better information can be found. However, the findings emphasize the central role of physicians in communicating with patients regarding HIV/AIDS and in acquiring knowledge, since consistent with prior research health care providers were positively associated with greater levels of knowledge about HIV/AIDS [13,14]. This finding support the importance of the health care provider’s role in initiating lines of communication regarding HIV/AIDS related problems. Moreover, two-thirds of the respondents desired to learn more about HIV/AIDS and this indicates that targeted interventions are needed to increase understanding also because health promotion programs are not regularly provided for this population.

The analysis of the results regarding the cited reasons for seeking VCT showed that the most commonly were for learning about their HIV sero-status and own or partners’ high risk sexual behaviors. This result is in line with previous findings [7-9,14].

While the current study has revealed important messages, there are a number of limitations that need to be taken into account in interpreting the results. A primary limitation of this study is that the design is a cross-sectional questionnaire study and, by evaluating the participants at one point of time, making it impossible to deduce causal relationships between socio-demographic and behavior characteristics and the different outcomes of testing. Secondly, the survey respondents may have been the more motivated subpopulation with those who were not interested abstaining from participation and participation bias is particularly likely, because it is conceivable that people who have strong negative attitudes may be the least inclined to complete the study questionnaire. Thus, the study achieved a high response rate and, therefore, we believe such participation bias if present was overall minimal. Thirdly, the study relied on sensitive information which may have limited honest responses, particularly on sexual and substance use behaviors. As a result, these findings may be subject to potential recall bias, participants may have been reluctant to reveal true risk behaviors thus underestimating the level of high-risk behaviors. To minimize such bias, the questionnaire was self-administered.

## Conclusions

In conclusion, this study suggest that the level of unsafe sexual behavior is considerable and the level of knowledge amongst this sample in general is poor. The findings have significant implications for health communication and interventions are required to develop initiatives with the aim of improving knowledge about HIV/AIDS.

## Competing interests

The authors declare that they have no competing interests.

## Authors’ contributions

GDG participated in the design of the study, contributed to the data analysis and interpretation; AS participated in the design of the study, contributed to the data analysis and interpretation; SM was responsible for the data collection; NC participated in the design of the study, was responsible for the data collection; IFA, the principal investigator, designed the study, was responsible for the data collection, statistical analysis and interpretation, and wrote the article. All authors have read and approved the final version of the manuscript.

## Pre-publication history

The pre-publication history for this paper can be accessed here:

http://www.biomedcentral.com/1471-2334/13/277/prepub
